# Supplementary material for: Plasmon-driven nanowire actuators for on-chip manipulation
Source: Nat Commun. 2021 Jan 15;12:385. doi: 10.1038/s41467-020-20683-2 (PMC7810692; doi:10.1038/s41467-020-20683-2)
Supplement: Supplementary file 1 — Supplementary Information [file 41467_2020_20683_MOESM1_ESM.pdf]

Supplementary Information for

Plasmon-driven nanowire actuators for on-chip manipulation

Shuangyi Linghu[1,†], Zhaoqi Gu[1,†], JinSheng Lu[2], Wei Fang[2], Zongyin Yang[3], Huakang Yu[4], Zhiyuan Li[4], Runlin Zhu[1], Jian Peng[1], Qiwen Zhan[1,5], Songlin Zhuang[1], Min Gu[6] and Fuxing Gu[1,*]

[1]Laboratory of Integrated Opto-Mechanics and Electronics, Shanghai Key Laboratory of Modern Optical System, Engineering Research Center of Optical Instrument and System (Ministry of Education), University of Shanghai for Science and Technology, Shanghai 200093, China

[2]State Key Laboratory of Modern Optical Instrumentation, College of Optical Science and Engineering, Zhejiang University, Hangzhou 310027, China

[3]Cambridge Graphene Centre, University of Cambridge, Cambridge CB3 0FA, UK.

[4]School of Physics and Optoelectronics, South China University of Technology, Guangzhou 510641, China

[5]Department of Electro-Optics and Photonics, University of Dayton, 300 College Park, Dayton, Ohio 45469, USA

[6]Centre for Artificial-Intelligence Nanophotonics, School of Optical-Electrical and Computer Engineering, University of Shanghai for Science and Technology, Shanghai, 200093, China.

†These authors contributed equally to this work.

*Correspondence and requests for materials should be addressed to F.G. (email: gufuxing@usst.edu.cn)

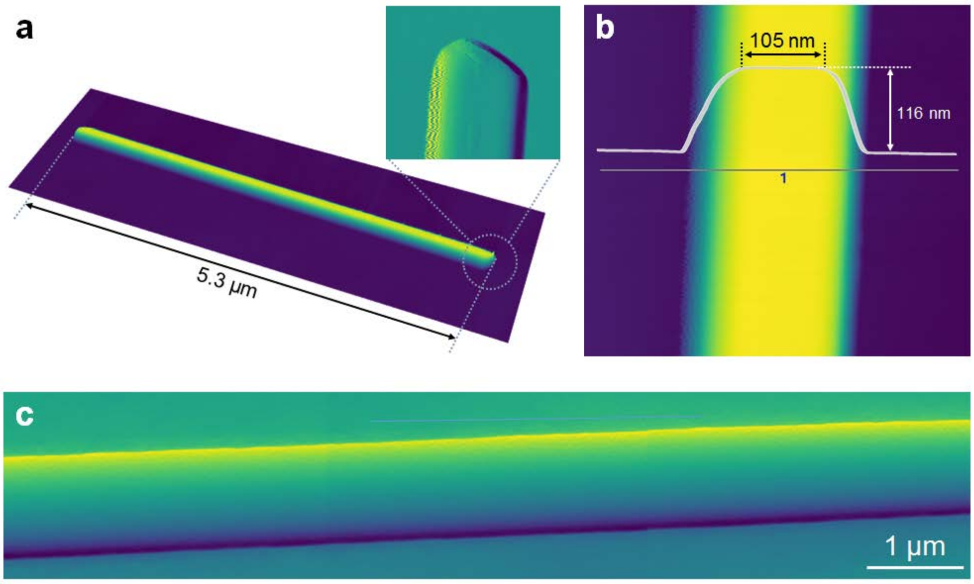

Supplementary Figure 1. Typical atomic force microscopy (AFM) images of an Au nanowire and a silica microfibre. (**a**) AFM image of an Au nanowire with L_{NW} = 5.3 μm. Insert shows the image of the Au nanowire tip with a rhombic shape, and we can also see that the surface of the nanowire is very smooth. (**b**) High-resolution image of the Au nanowire in (**a**). The AFM line scan from the region indicated by the dashed line shows the nanowire cross section is approximately rectangular, with a width of 105 nm and a thickness of 116 nm. (**c**) High-resolution AFM image of a typical silica microfibre, which is stitched from three AFM scanned images. The measured surface arithmetical mean roughness (*R*a) is 280 pm for the Au nanowire and is 140 pm for the silica microfibre.

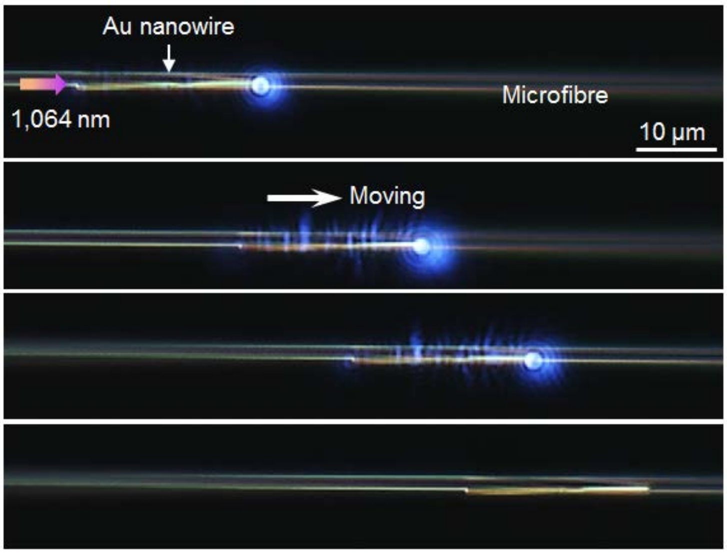

Supplementary Figure 2. Movement of an Au nanowire (L_{NW} = 22.4 μm) on a silica microfibre (D_{fibre} = 1.9 μm), actuated by a 1,064 nm pulsed-laser with average power of 460 μW. Note that under the 1,064-nm laser actuation from the left side, the Au nanowire moved to the right.

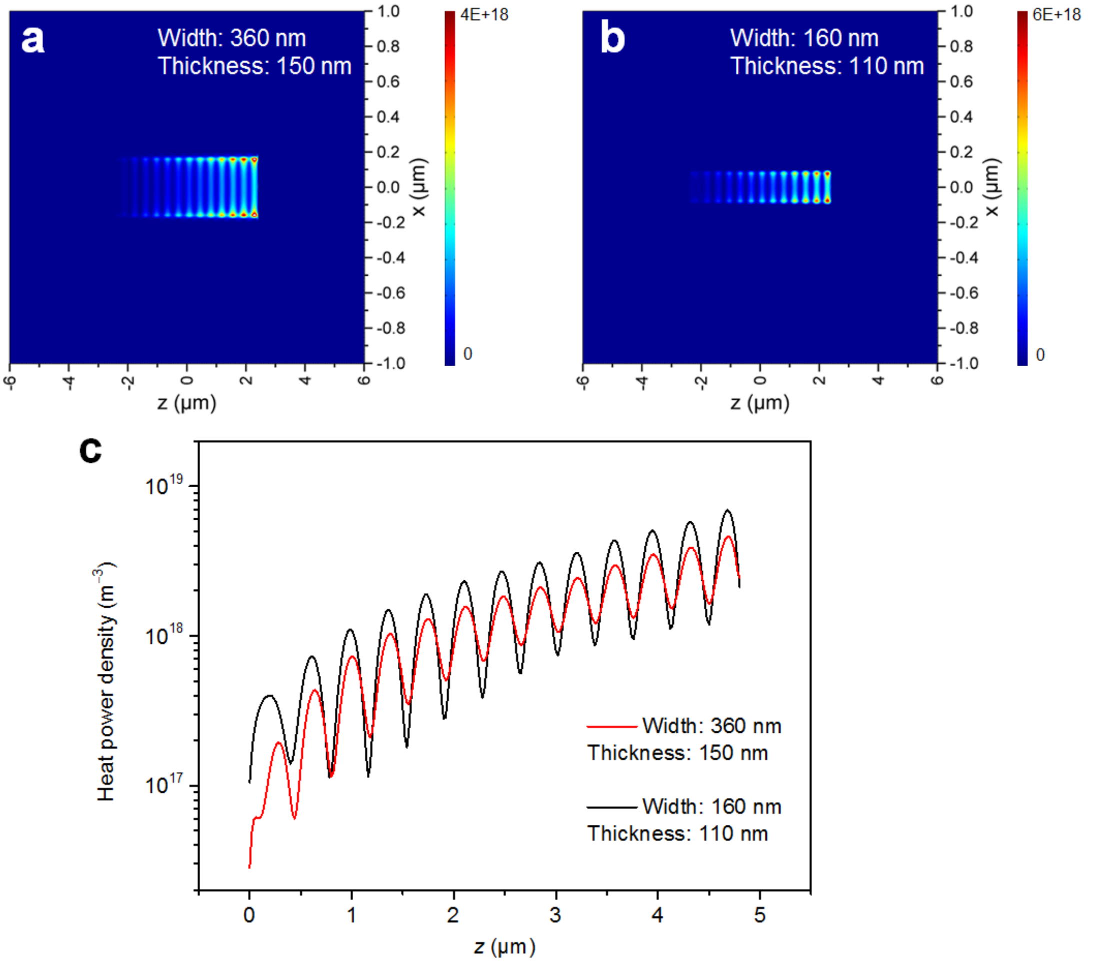

Supplementary Figure 3. Influence of the nanowire cross-sections on the moving speeds. We experimentally tested two Au nanowires with different cross-sections but approximately the same lengths (L_{NW} = 4.8 μm), which were driven by the same 1,064-nm light power (3.1 μW) and on the same microfibre (D_{fibre} = 2.1 μm). It shows that the moving speed (10.9 μm s^{-1}) of the thick nanowire (**a**: 360 nm × 150 nm in width and thickness) was lower than that (18.9 μm s^{-1}) of the thin nanowire (**b**: 160 nm × 110 nm in width and thickness). (**a**) and (**b**) shows the calculated spatial distributions of heat power density on the Au nanowire bottom surfaces. (**c**) Heat power density value along the sideline (*z* direction) of the nanowire bottom surfaces. It can be observed that the maximum heat densities in two nanowires are on the same order of magnitude. The higher moving speed of the thinner nanowire can be attributed to its smaller adhesion force to the microfibre surface.

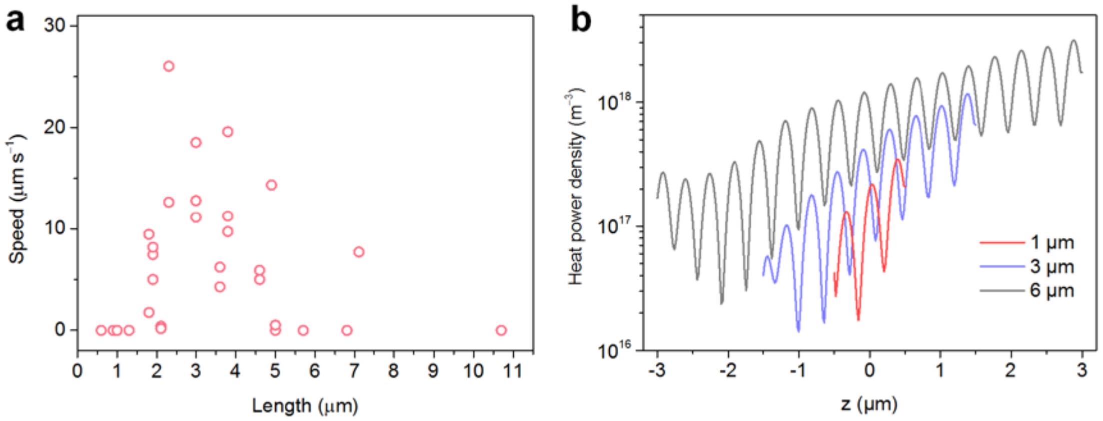

Supplementary Figure 4. Experimental statistics of the dependence of the nanowire lengths on the moving speeds. (**a**) Dependence of the nanowire lengths on the moving speeds. The used nanowires were placed on a same microfibre with a diameter of 2.3 μm, and the input power of the 1,064-nm nanosecond laser was maintained at ~0.8 μW. (**b**) Heat power density value along the sideline of the nanowire bottom surface with different lengths. We can see that for 1-μm-length nanowire, the induced heat power density in the 1-μm-length nanowire is one order of magnitude smaller than those of other two nanowires with lengths of 2.0 μm and 6.0 μm.

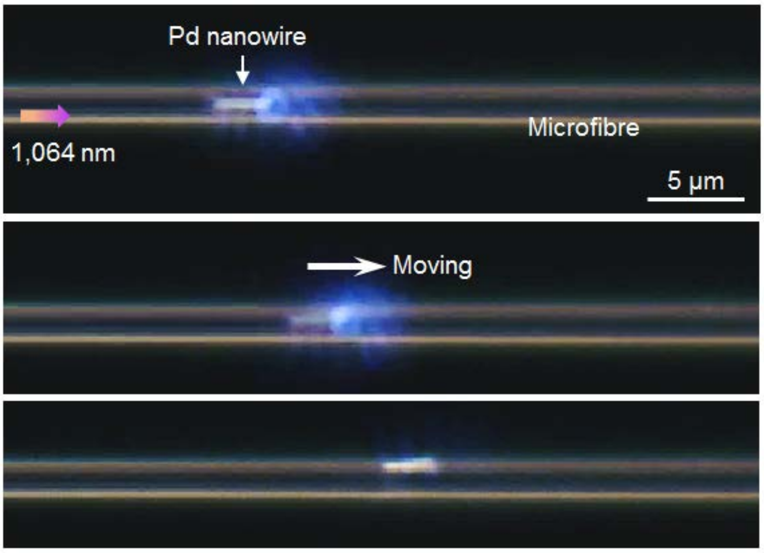

Supplementary Figure 5. Movement of a Pd nanowire (L_{NW} = 2.8 μm) on a silica microfibre (D_{fibre} = 2.0 μm) actuated by a 1,064 nm pulsed-laser with average power of 200 μW. Note that under the 1,064-nm laser actuation from the left side, the Pd nanowire moved to the right.

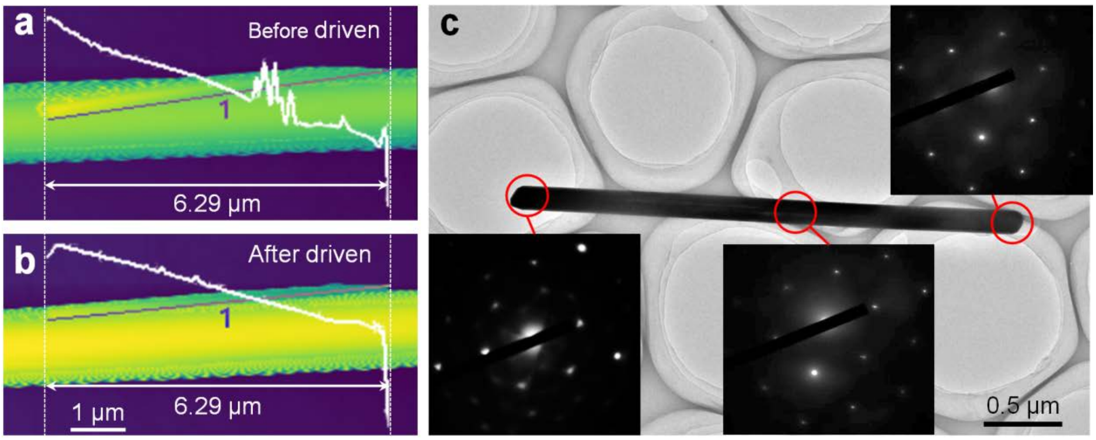

Supplementary Figure 6. Intrinsic characteristics of the Au nanowires driven by the 1,064-nm nanosecond pulsed laser. Length characteristics of an Au nanowire (width: 170 nm, thickness: 120 nm, and length: ~6.3 μm) (**a**) before and (**b**) after driven to move a distance of 3.5 μm by a 1,064-nm nanosecond pulsed laser (light power: ~6.0 μW) on a microfibre (D_{fibre} = 1.5 μm), which were characterized using a high-resolution atomic force microscope (AFM, Asylum Research Cypher Oxford Instruments). The AFM scanning results in (**a**) and (**b**) show that the nanowire has no detectable change in the length before and after the SPP driven. (**c**) Crystal characteristics of an Au nanowire after driven by the 1,064-nm nanosecond pulsed laser, which was characterized using a high-resolution transmission electron microscopy (TEM). The regular lattice diffraction spots of its electron diffraction patterns at the front, middle and back of the nanowire, indicate a single crystal behavior of the nanowire.

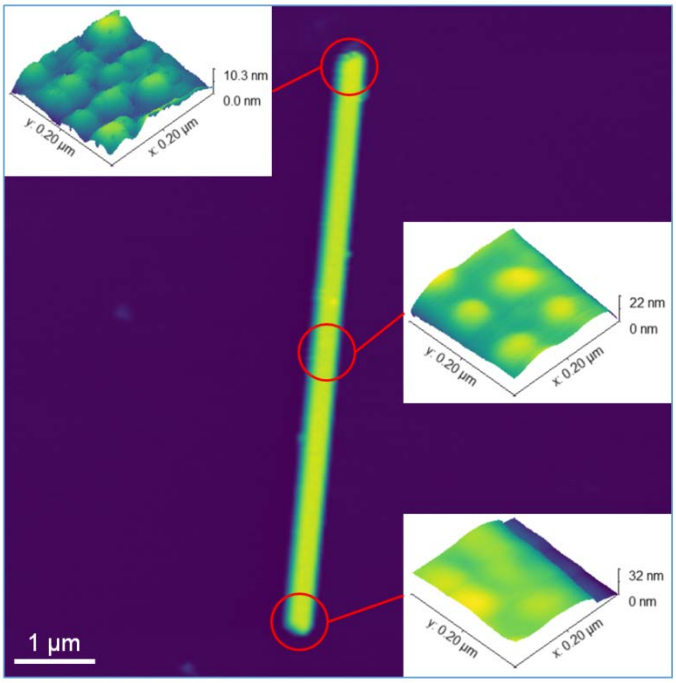

Supplementary Figure 7. AFM scanning results of an Au nanowire driven by the 1,035-nm-wavelength picosecond pulses. The used Au nanowire was driven by the

1,035-nm-wavelength picosecond pulses (light power: ~1.0 μW) on a microfibre (D_{fibre} = 1.7 μm). From the scanning results at the front, middle and back of the nanowire, we can see obvious small dots on the surface. Compared with the smooth surface before the laser driving (typical image shown in **Supplementary Figure 1**), the increased roughness suggest a recrystallization or reconstruction effect on the surface.

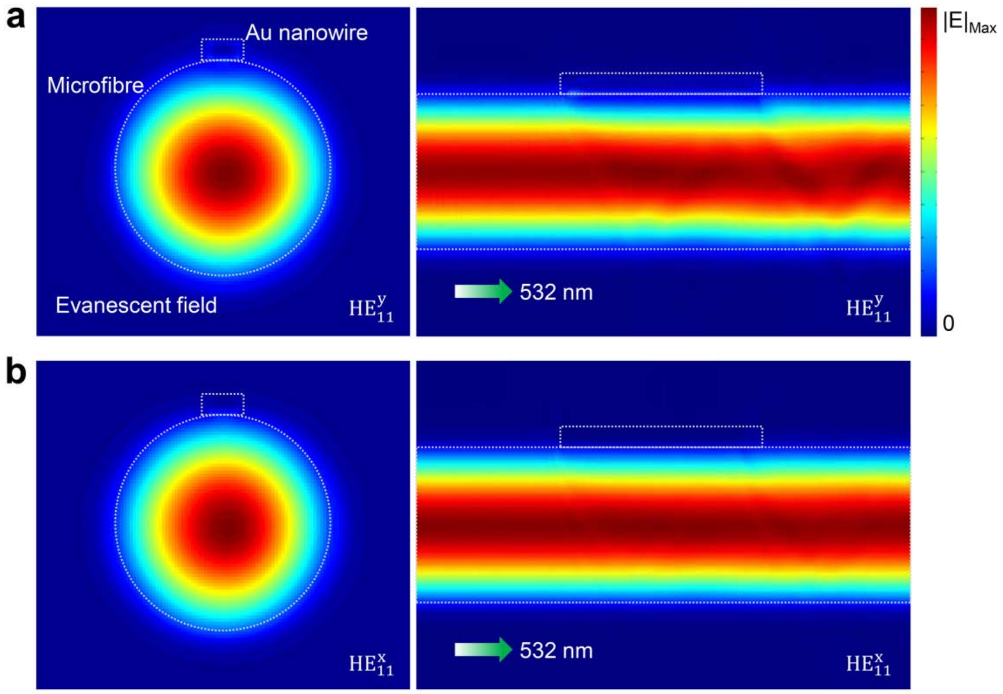

Supplementary Figure 8. Simulated electric field distributions of the nanowire-microfibre system at 532 nm. Simulated excitation results by (**a**) HE_{11}^{y} fundamental mode and (**b**) HE_{11}^{x} fundamental mode of guided 532-nm light in the microfibre. Upper and lower images show the distributions on the cross planes of the nanowire-microfibre system along its radial and axis directions, respectively. The Au nanowire has a thickness of 100 nm, a width of 200 nm, and a length of 6.0 μm, and the microfibre has a D_{fibre} of 1.8 μm. For both modes, no significant excited SPPs are observed around the nanowire–microfibre interface.

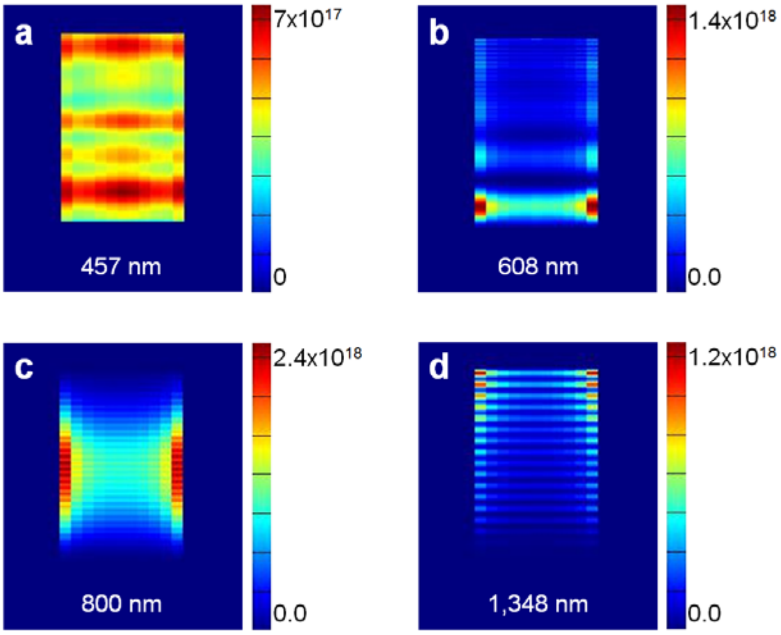

Supplementary Figure 9. Heat power density distributions on the bottom surface of an Au nanowire, excited by the guided HE_{11}^{y} modes in the microfibre with different wavelengths. The Au nanowire has a thickness of 100 nm, a width of 200 nm, and a length of 6.0 μm, and the microfibre has a D_{fibre} of 1.8 μm. It can be found that the light in longer wavelength ranges can induce dominant absorptance due to the well excited SPPs, and the heat power density also shows gradient distributions along the nanowire bottom.

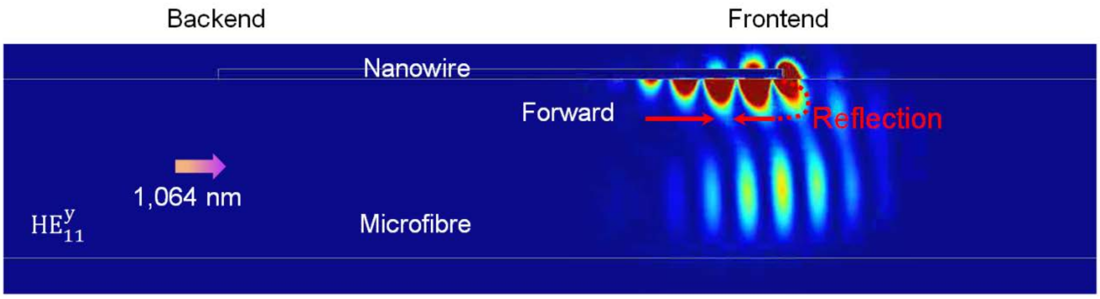

Supplementary Figure 10. Formation of the SPP standing wave along the nanowire–microfibre interface at 1,064 nm, by using an FDTD method. When reaching the nanowire frontend (relative to the guided light direction), the accumulated forward propagating SPPs will be reflected by the nanowire frontend and propagate backward, thus the forward and the backward SPPs overlap to form a standing wave. Due to the existence of propagating loss, the intensity of the SPP standing wave reaches the maximum at the nanowire frontend, and quickly decays toward the backend. **Supplementary Movie 2** shows the real time recording of this formation dynamic process.

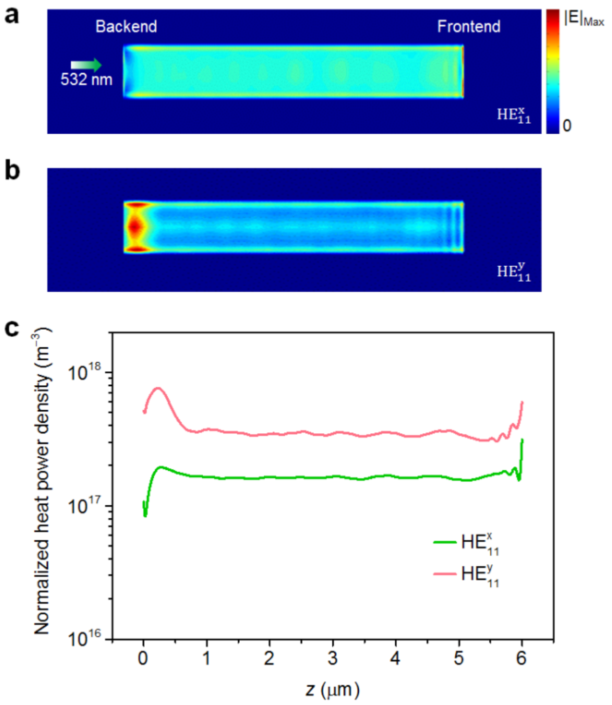

Supplementary Figure 11. Simulated heating effect in an Au nanowire excited by the guided 532-nm light. (a), (b) Spatial distributions of heat power density on the Au nanowire bottom surface induced by the 532 nm (**a**) $HE_{11}{}^{x}$ modes and (**b**) $HE_{11}{}^{y}$ modes guided in the microfibre. **c** Heat power density value along the sideline (*z* direction) of the nanowire bottom surface. The Au nanowire has a thickness of 100 nm, a width of 200 nm, and a length of 6.0 μm, and the supported microfibre has a D_{fibre} of 1.8 μm. We can see that for the $HE_{11}{}^{x}$ modes, the heat power density is approximately evenly distributed along the nanowire bottom, so that the nanowire cannot be driven along the microfibre. For the $HE_{11}{}^{y}$ modes, the heat power density is highly concentrated around the nanowire frontend, thus the nanowire can be driven.

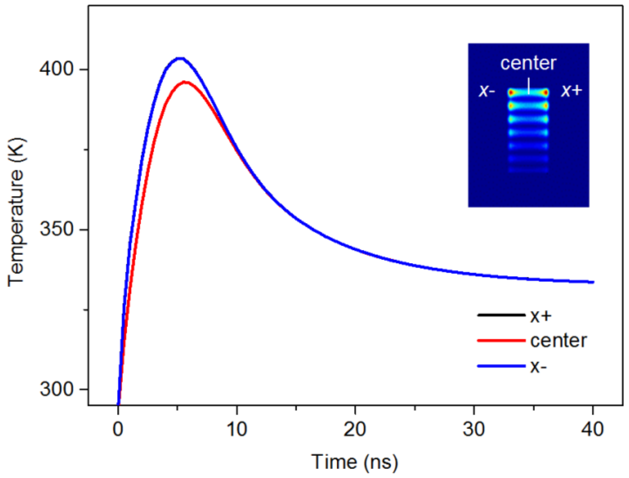

Supplementary Figure 12. Calculated time-dependent transient temperature change at the bottom of the Au nanowire shown in Fig. 4c (in main text). Inset: heat power density distribution at the nanowire bottom, which is denoted with *x*+ side, *x*- side, and center.

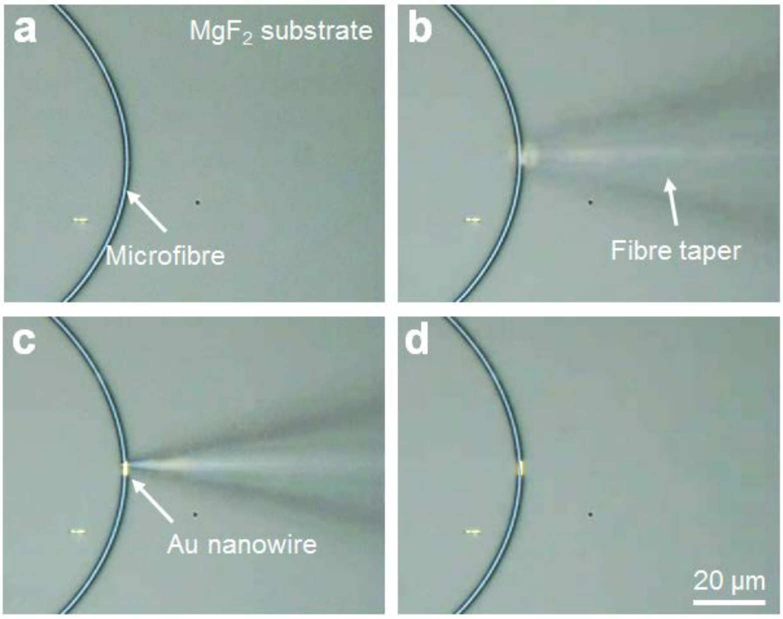

Supplementary Figure 13. Schematic of micromanipulation process of picking up an Au nanowire by using a fibre taper with a sharp tip. (**a**) A silica microfibre placed on a MgF_2 substrate. (**b**) Vertical Au nanowires were grown on the *c*-cut sapphire substrate. Under an optical microscope equipped with long-working distance objectives, a fibre taper with a sharp tip was mounted on a triple-axis micromanipulator (M-462, Newport) and then used to pick up the Au nanowire[1]. (**c**) Closely contact the Au nanowire with the surface of the microfibre. (**d**) Due to the van der Waals and electrostatic attraction, the Au nanowire was placed on the microfibre.

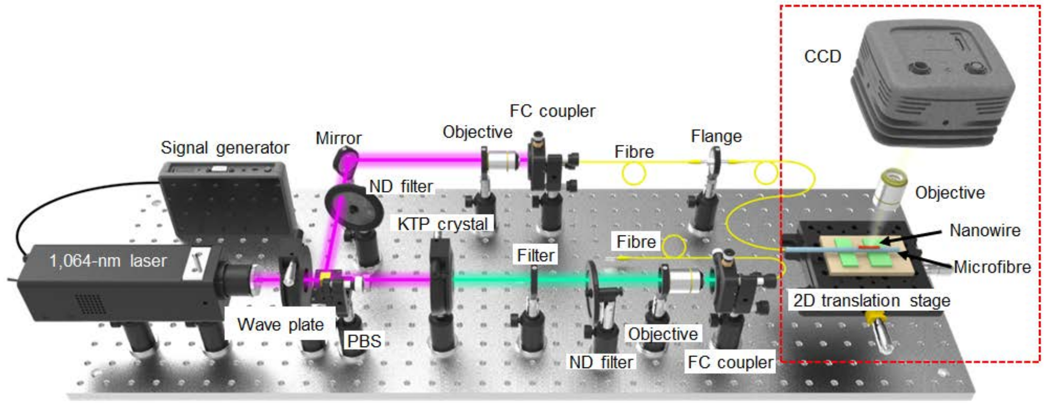

Supplementary Figure 14. Schematic diagram of an experimental setup for optical characterization of the metal nanowire movement. Mirror: silver mirror; ND filter: neutral density filter; PBS: polarizing beam splitter. The red dotted box shows a brief structure of an optical microscope (Nikon Eclipse ME600). The metal nanowires on silica microfibres used in this work were placed on a 2D translation stage under a long-working distance objective. A CCD camera (DS-Fi1, Nikon) was used for monitoring and imaging.

Supplementary Note 1. Measurement of the adhesion force between the Au nanowire and silica microfibre

We assume that the Au nanowire is uniform in diameter and smooth in the sidewall. The adhesion force is non-directional, and it prevents the Au nanowire from leaving the microfibre surface in any direction. In order to measure the adhesion force, we used a microfibre probe protruded out of a MgF_2 substrate (denoted as M1) to touch an Au nanowire, which was also suspended in the air from another MgF_2 substrate (denoted as M2), as shown in **Supplementary Figure 15**. The M1 substrate with a microfibre was precisely driven by a precision 3D piezoelectric stage. By moving the M1 substrate, the axis of the nanowire and the microfibre was spatially in contact and aligned with an overlap of 4 μm, which is a typical length of Au nanowires in our work (**Supplementary Figure 15a**). Then by moving the M1 substrate in the *y*-direction, the adhesion force between the Au nanowire and silica microfibre in the axial direction, which acts as the friction force, pulled the microfibre and caused the microfibre to bend (**Supplementary Figure 15b**). When the force applied on the microfibre due to bending was greater than the friction force, the nanowire was detached from the microfibre and the microfibre restores to its initial position (**Supplementary Figure 15c**).

We can build an elastic deformation model to analyze the adhesion force between Au nanowire and silica microfibre. The adhesion force in the azimuthal direction (i.e. the friction force) is equal to the maximum bending force of the nanowire, which can be calculated from the elastic deformation model of the cantilever (**Supplementary Figure 15d**). The point load (P) and the adhesion force (F_{ad}) can be calculated by using the following formula:

$$P = \frac{3\delta GI}{l^3} \quad \text{(S1)},$$

$$F_{ad} = \frac{P}{\sin\theta} \quad \text{(S2)},$$

$$\delta = P\left(\frac{lx^2}{2GI} - \frac{x^3}{6GI}\right) \quad \text{(S3)},$$

$$\theta = \delta'_x = P\left(\frac{lx}{GI} - \frac{x^2}{2GI}\right) \quad \text{(S4)},$$

$$I = \frac{1}{2}I_\rho = \frac{1}{2}\int_A \rho^2 dS = \frac{\pi}{64}D^4 \quad \text{(S5)},$$

where δ is the deflection; δ_{max} (see inset in **Supplementary Figure 15d**) is the maximum deflection at the free end when $x = l$); θ is the angle of deflection; P is the point load at the site of x; G is the Young's modulus of the microfibre (silica); I is the second moment of area; ρ is the distance to the center of the cross-section; D is the diameter of the microfibre; l is the length of the cantilever, and in our measurement, $x = l$.

The microfibre has a certain angle θ with the axis of the nanowire, so the adhesion needs to be corrected according to θ, 10 ~ 14 degrees. We select the middle value of 12 degrees, and then we obtain the values of P and F_{ad} to be 0.4 μN and 1.8 μN, respectively. Furthermore, we

substitute P into this eq.S2 and get the value θ of 12.35 degrees, which is within a reasonable range.

We also use the formula of van der Waals force to estimate this adhesion force theoretically. The van der Waals force F_v for the cylinder-planar surface can be estimated as[2,3]

$$F_v = \frac{AL\sqrt{D}}{16z^{5/2}} \quad \text{(S6)},$$

where A is the Hamaker constant; D is the diameter of the cylinder; z is the separation distance between the cylinder and the planar surface; L is the length of the cylinder contacted with the planar surface. Then we choose the separation distance between microfibre and nanowire to be 1 nm, and the van der Waals force is calculated to be 1.2 μN, which is roughly consistent with the measured force (with the surface arithmetical mean roughness by AFM, the force can be calculated by the eq. S6. The typical balance gap for gravitational and repulsive force is 0.2 nm for the metal[4].

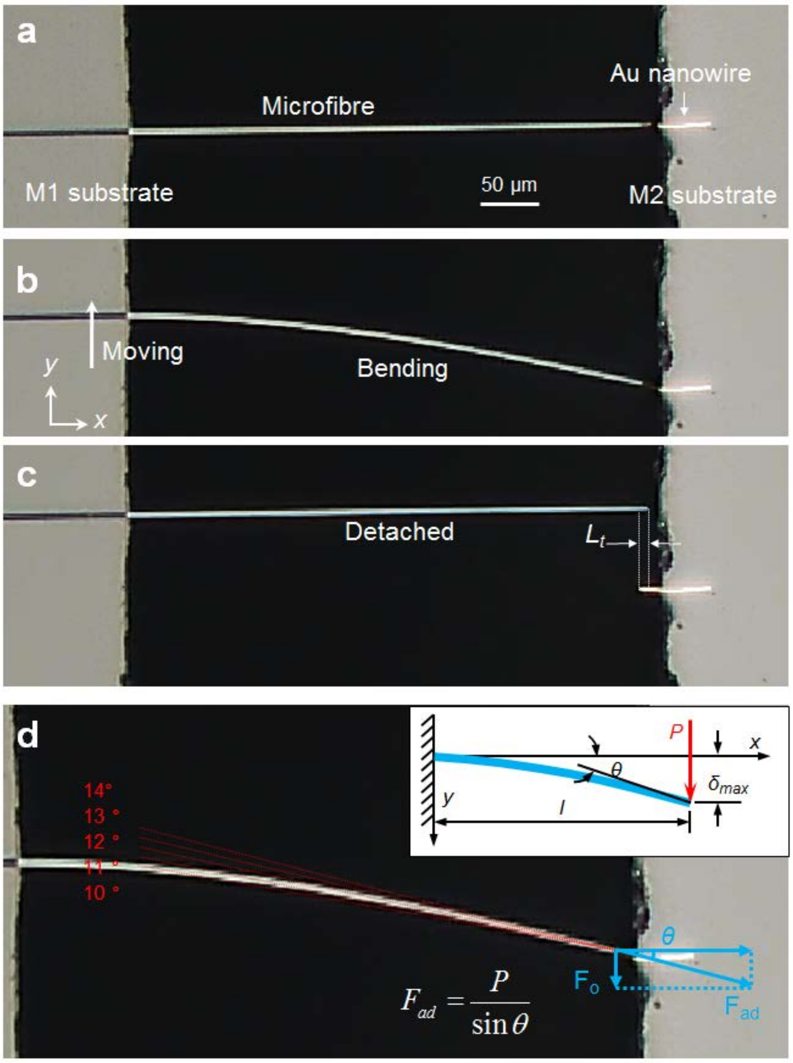

Supplementary Figure 15. Measurement of the adhesion force between the Au nanowire and silica microfibre. (**a**–**c**) Experimental procedures to measure the adhesion force. (**d**) Enlarged view of the microfibre/nanowire structure in (**b**). The inset in (**d**) shows the schematic illustration for calculating the forces.

Supplementary Note 2. Numerical simulations of the electric field and the heating power density

We use a 3D FDTD (Finite-Difference Time-Domain) method (a commercial software Lumerical FDTD solution v8.15) to calculate the electric field distributions of our system. The Au nanowire is chosen to be a rectangle cross-section, with the width 200 nm, and the thickness 100 nm, and its length varies according to the different conditions. Furthermore, in the simulation, we assume that the Au nanowire is uniform in refractive index and smooth in the sidewall as well. The refractive index of background is set to be 1, and the index of Au and SiO_2 are set to be 0.389 + 2.11i, 1.46 at 532 nm, and 0.106 + 6.82i, 1.45 at 1,064 nm, respectively. We use a grid size (*dx*, 20 nm; *dy*, 10 nm; *dz*, 20 nm) for the Au nanowire-silica microfibre structure, for a higher precision is needed in the direction perpendicular to the surface of microfibre, and set the gap between the Au nanowire and microfibre as 1 nm. In experiments, the guided modes in the microfibre were quasi-circular polarized which had both E_x and E_y components, but we find the mode E_x has hardly effect to drive nanowire, so we consider the fundamental mode E_y in simulations.

From the results of FDTD simulations, the electric field distribution in the Au nanowire can be extracted and the heat power volume density $Q_d\ (r,\lambda)$ ($W\ m^{-3}$) can be calculated as

$$Q_d(r,\lambda) = \frac{1}{2}\varepsilon_0 \omega \,\mathrm{Im}(\varepsilon_r)\left|\mathbf{E}(\mathbf{r},\lambda)\right|^2 \qquad \text{(S7)},$$

where $\boldsymbol{\varepsilon_0}$ is the vacuum permittivity; $\boldsymbol{\omega}$ is the frequency of light source; $\mathrm{Im}(\varepsilon_r)$ is the imaginary part of the relative permittivity of the gold. $\mathbf{E}(\mathbf{r},\lambda)$ is the electric field intensity with a specific wavelength λ in the Au nanowire.

The light absorptance spectrum (Fig.2c in the main body) can be calculated using volume integral of $Q_d(\mathbf{r},\lambda)$ (eq.S7), which is not included scattering. We also calculate the heat power density distributions on the bottom surface of the Au nanowire with several specific wavelengths. The heat source of a nanosecond light pulse in the experiment can be described by a Gaussian pulse function as

$$Q_d(x,y,z,t) = P_0 \cdot Am \cdot \frac{M}{\sqrt{\pi}\tau}\exp\left[-\frac{(t-t_0)^2}{\tau^2}\right]\exp\left[-\frac{(x-x_0)^2}{s_x^2}-\frac{(y-y_0)^2}{s_y^2}-\frac{(z-z_0)^2}{s_z^2}\right] \text{(S8)},$$

where M is the period of the pulsed laser source; $\boldsymbol{\tau}$ ns is the time constant of the light pulse; t_0 is the time delay of the pulse peak. P_0 is the input time average power of the pulsed laser; Am is the normalized power density from the FDTD simulation; $1/s_x$, $1/s_y$, and $1/s_z$ are the damping factor in three spatial components. Note that $Q_d(\mathbf{r})$ refers to the heat power volume density with a unit of W/m^3, but we make it normalized to the source power. So we get the unit m^{-3} in Fig. 2d and 2e (in the main text).

The transient temperature $T(\mathbf{r}, t)$ can be calculated using the heat conduction equation:

$$C\frac{\partial T(\mathbf{r},t)}{\partial t} + \nabla\cdot(k\nabla T(\mathbf{r},t)) = Q_d(\mathbf{r},t) \qquad \text{(S9)},$$

where C and k are the thermal capacity and thermal conductivity, respectively. COMSOL Multiphysics software is used to solve this equation and the reference temperature is 293.15 K. For example, we choose the length of the Au nanowire to be 2.3 μm with the source light 1,064

nm in FDTD simulation, and the simulation suggests that the power source points are more than one and symmetrically distributed. Then we choose two points (x+, x-) with the highest intensity as heat sources in the COMSOL simulation, and show transient temperatures of three points, x+, x-, center (midpoint between x+ and x-, **Supplementary Figure 12** and inset). The peaks of the transient temperatures of the center and side points are calculated to be about 396.6 K, 403.8 K, respectively.

The acoustic wave equation with an external force resulting from thermoelastic deformation mechanisms (in COMSOL) can be described as:

$$\rho \frac{\partial^2 u}{\partial t^2} = \nabla \cdot s + F_v \qquad \text{(S10)},$$

where u is the displacement vector; s is the total stress; F_v is the body load (we set none).

The expansion of the Au nanowires in thickness during one pulse can be calculated as

$$\delta d = \alpha \times \Delta T \times d \qquad \text{(S11)},$$

where α, ΔT, and d are thermal expansion coefficient, temperature increment, and thickness of the Au nanowire, respectively. The thickness of the Au nanowire is selected to be half because the generated Lamb wave is symmetric at first.

Table S1 Definitions and values of parameters used in this work.

Parameter	Definition	Value/unit
M	Period of the pulsed-laser source	250 μs
τ	Time constant of the light pulse	5 ns
t_0	Time delay of the pulse peak	1.5 ns
$1/s_x$	Spatial damping factor (for 2.3 μm)	5.50×10^7 m^{-1}
$1/s_y$		8.26×10^7 m^{-1}
$1/s_z$		8.83×10^6 m^{-1}
$1/\boldsymbol{s}_x$	Spatial damping factor (for 6.0 μm) in the main text	5.50×10^7 m^{-1}
$1/s_y$		9.71×10^7 m^{-1}
$1/s_z$		9.17×10^6 m^{-1}
C	Thermal capacity of Au nanowire	120 J/(kg·K)
k	Thermal conductivity of the Au nanowire	150 W/(m·K)
α	Thermal expansion coefficient of Au nanowire	31.5 μ/K
P_0	Input time average power	0.347 mW
Am	Normalized power density from the FDTD simulation(for 2.3 μm)	1.29×10^{18} m^{-3}
Am	Normalized power density from the FDTD simulation(for 6.0 μm) in the main text	8.39×10^{18} m^{-3}
G	Young’s modulus	73.1 GPa
l	Length of the microfibre	229.7 μm
A	Hamaker constant	1.12×10^{-19} J
L	Contacted length	4.0 μm
z	Separation distance	1 nm
D	Diameter of the microfibre	1.9 μm

Supplementary Note 3. Influence of the picosecond pulses on the nanowire movement

Because our mechanism is based on SPP induced heating effect, we investigate the influence of the pulse duration on the nanowire movement, by theoretical calculations of the pulsed laser induced transient temperature in the nanowires. For picosecond pulses, we used the eq.S8 and eq.S10 to simulate the processes of pulses heating and surface acoustic wave (using shorter time steps and smaller time delays of pulse peak t_0), and compared the results with that of the 1 ns pulse.

For short pulses less than 0.5 ns, the nanowire has obvious unbalanced spatial-temporal temperature response between the side and the center of the frontend. In **Supplementary Figure 16**, the dashed and solid lines show the calculated time-dependent transient temperature changes at the side and center point respectively. At the very beginning of the light absorption, the heat power density at the nanowire bottom shows an unbalanced distribution for these short pulses, in which the temperature difference between the center and side points is very large. For example, the temperature differences between the center and side points at the nanowire bottom are 760 K, 1170 K and 590 K for time durations of 6 ps, 50 ps and 0.2 ns respectively (**Supplementary Figure 16**), but this difference in **Supplementary Figure 12** is much smaller by using the nanosecond pulses, only 7 K. Such high temperature difference cannot be ignored. We think that in such an ultra-short time scale (less than 0.2 ns), the heat transfer from the side point to the center point could not complete, and the highly concentrated heat at the side point may damage the nanowire lattice. AFM scans reveal that the surface roughness of as-driven nanowires has increased a lot, suggesting a recrystallization or reconstruction effect on the nanowire surface. For femtosecond pulses, the peak power of the pulses is much higher and will induce more unbalanced spatial-temporal temperature responses. Experimentally the phenomena of driving nanowires with femtosecond pulses (~600 fs) and picosecond pulses (~6 ps) were very similar.

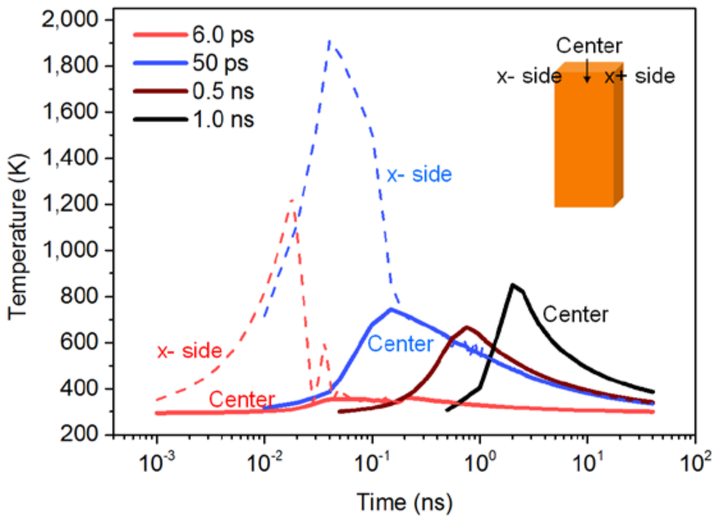

Supplementary Figure 16. Calculated time-dependent transient temperature changes at the bottom of the Au nanowire with different duration less than 1 ns. The solid and dashed lines represent time-dependent transient temperature change at the center and side points (x+, x-).

Supplementary References

1. Gu. F. et al. Single-crystal Pd and its alloy nanowires for plasmon propagation and highly sensitive hydrogen detection. *Adv. Opt. Mater.* **2**, 189–196 (2014).
2. Lu. J. et al. Nanoscale lamb wave-driven motors in nonliquid environments. *Sci. Adv.* **5**, eaau8271 (2019).
3. Hamaker, H. C. The london–van der waals attraction between spherical particles. *Physica* **4**, 1058–1072 (1937).
4. Alvarez, S. A cartography of the van der waals territories. *Dalton Trans.* **42**, 8617–8636 (2013).
