# Supplementary material for: Plasmon-driven nanowire actuators for on-chip manipulation
Source: Nat Commun. 2021 Jan 15;12:385. doi: 10.1038/s41467-020-20683-2 (PMC7810692; doi:10.1038/s41467-020-20683-2)
Supplement: Supplementary file 3 — Description of Additional Supplementary Files [file 41467_2020_20683_MOESM3_ESM.docx]

**Description of Additional Supplementary Files**

**Supplementary Movie 1:** Nanowire movement driven by the 664-nm long-pass filtered

supercontinuum source.

**Supplementary Movie 2:** Formation of the SPP standing wave.

**Supplementary Movie 3:** Propagation of a symmetric plate wave in a nanowire.

**Supplementary Movie 4:** The phenomenon of self-parallel parking.

**Supplementary Movie 5:** Transport of an Au nanowire along a C-shaped curved microfiber.
